# Sex differences in myocardial injury after non-cardiac surgery and postoperative mortality

**DOI:** 10.1186/s13741-023-00294-3

**Published:** 2023-03-16

**Authors:** Ji-Hye Kwon, Jungchan Park, Seung-Hwa Lee, Cheol Won Hyun, Jihoon Kim, Kwangmo Yang, Jeong Jin Min, Jong Hwan Lee, Sangmin Maria Lee, Jin-ho Choi, Sang-Chol Lee, Hyeon-Cheol Gwon, Sukyoung Her, Kyunga Kim, Joonghyun Ahn

**Affiliations:** 1 Department of Anesthesiology and Pain Medicine, Samsung Medical Center, Sungkyunkwan University School of Medicine, Seoul, South Korea; 2 Wiltse Memorial Hospital, Suwon, South Korea; 3 Division of Cardiology, Department of Medicine, Heart Vascular Stroke Institute, Samsung Medical Center, Sungkyunkwan University School of Medicine, 81 Irwon-Ro, Gangnam-Gu, Seoul, 06351 Korea; 4 Center for Health Promotion, Samsung Medical Center, Sungkyunkwan University School of Medicine, Seoul, South Korea; 5 Statistics and Data Center, Research Institute for Future Medicine, Samsung Medical Center, Seoul, South Korea; 6 Department of Digital Health, SAIHST, Sungkyunkwan University, Seoul, South Korea

**Keywords:** Sex difference, Non-cardiac surgery, Cardiac troponin, Mortality

## Abstract

**Background:**

Myocardial injury after non-cardiac surgery (MINS) has recently been accepted as a predictor of mortality. However, sex differences in the incidence of MINS and survival thereafter are not fully understood. This study aimed to compare the incidence of MINS and mortality among male and female patients.

**Methods:**

This single-center study was conducted using the database of a large tertiary referral hospital. Consecutive patients with cardiac troponin (cTn) detected within 30 days after non-cardiac surgery performed between January 2010 and June 2019 were grouped according to sex. The incidence of MINS and mortality of patients with MINS were compared between men and women.

**Results:**

Of the 33,311 patients, 18,546 (55.7%) were men and 14,765 (44.3%) were women. In a multivariable analysis, women showed a significantly lower incidence of MINS than did men (17.9% vs. 14.2%; odds ratio, 0.76; 95% confidence interval [CI], 0.71–0.81; *P* < 0.001). In patients with MINS, the propensity-score-matched analysis showed that 30-day mortality did not differ according to sex, but mortality in females was significantly lower than that in males during the overall follow-up (33.0% vs. 25.7%; hazard ratio, 0.75; 95% CI, 0.66–0.84; *P* < 0.001).

**Conclusion:**

The incidence of MINS was lower in women than in men. In patients with MINS, female sex may be associated with a survival benefit. Further studies are needed to confirm these findings.

**Supplementary Information:**

The online version contains supplementary material available at 10.1186/s13741-023-00294-3.

## Background

Sex-related differences in the myocardial response to ischemia and reperfusion injury and the intrinsic differences between the male and female myocardium have been documented (Schwarzenberger et al. 2003), and their prognostic impact in various clinical settings has been speculated. The number of relevant studies on the cardiovascular system has grown exponentially over the past 20 years, reflecting the importance of this topic and the urgent need to better understand sex as a determinant of outcomes. However, the influence of sex on coronary artery disease remains controversial. Women were initially demonstrated to have a worse prognosis in coronary heart disease (Rosengren et al. 2001; Vaccarino et al. 1999), but other studies disputed these conclusions by adjusting for differences in comorbidities (Berg et al. 2017; Kovacic et al. 2012; Malenka et al. 2002). Although uncertainty remains, these studies indicated the existence of sex-related differences in cardiovascular disease and the need for specific therapeutic strategies according to sex for relevant pathologies.

In the 4th universal definition of myocardial infarction (MI), an isolated elevation of cardiac troponin (cTn) above the 99th percentile upper reference limit (URL) of any assay was defined as myocardial injury regardless of the presence of ischemic symptoms (Thygesen et al. 2018). Large observational studies have demonstrated that myocardial injury after non-cardiac surgery (MINS), defined as at least one report of cTn elevation above the 99th percentile URL within 30 days after surgery, as a result of myocardial ischemia, without the presence of ischemic symptoms, was associated with increased mortality for up to 2 years after surgery (Devereaux et al. 2011; Noordzij et al. 2015; Puelacher et al. 2018; van Waes et al. 2013; Writing Committee for the V S I et al. 2017). Despite ongoing debate on a causal relationship with mortality, MINS is widely considered as a strong predictor and the leading cause of postoperative mortality (Beattie et al. 2018; Botto et al. 2014). However, the clinical impact of sex on MINS has not been fully investigated. Therefore, we aimed to compare the incidence of MINS according to sex, in addition to evaluating whether sex differences affect mortality following MINS in the short and long term.

## Methods

We extracted all the data for this study from the Samsung Medical Center Troponin in Non-cardiac Operation (SMC-TINCO, KCT 0004244) registry, which is a large single-center cohort of de-identified patients. As all data for the registry were initially extracted in a de-identified form, the Institutional Review Board of the Samsung Medical Center waived the need for approval for this study and the requirement for written informed consent for access to the registry (SMC 2019–08-048). This study was conducted according to the principles of the Declaration of Helsinki and is reported following the Strengthening the Reporting of Observational Studies in Epidemiology guidelines.

### Data curation

We used the “Clinical Data Warehouse Darwin-C” of the Samsung Medical Center to extract raw data from the registry. This is an electronic system that was invented for investigators to search and retrieve de-identified medical records from the institutional electronic archive system. The accuracy and quality of data, especially relating to clinical record entries with potential for human error, were improved with this system. The study outcomes were MINS, mortalities, and length of hospital, which could be curated without human error using this system. After extracting data from the preoperative evaluation sheets, the patients’ baseline characteristics were presented in a standardized form by independent investigators who were blinded to mortality or cTn I levels.

### Study population and study endpoints

Between January 2010 and June 2019, a total of 265,562 consecutive patients who underwent non-cardiac surgery at the Samsung Medical Center, Seoul, Korea, and 43,019 patients (16.2% of the entire non-cardiac surgery population) who had measurable cTn I levels within 30 days after non-cardiac surgery were enrolled in the SMC-TINCO registry. From this registry, we excluded patients who were less than 18 years old, did not have postoperative cTn I data, or received cardiac massage before the diagnosis of MINS. Additionally, sex-specific procedures, that is, reproductive organ surgery such as obstetric and gynecologic surgery for women or prostate surgery for men were excluded from the analysis because these procedures can be performed either for men or women. After exclusions, a total of 33,311 patients were enrolled in this study and divided into two groups according to biological sex at birth. Of note, there were no patients with reassignment of biological sex in our database. The primary outcome was the incidence of MINS, and secondary outcomes included in-hospital mortality, mortality within 30 days and 1 year, overall follow-ups, and duration of hospital stay. After selecting patients with MINS, the difference in mortality according to sex was evaluated.

### Definitions

MINS was defined as elevation of cTn above the 99th percentile URL within 30 days after surgery, and elevations related to non-ischemic causes, including atrial fibrillation, cardioversion, pulmonary embolism, sepsis, or chronic elevation, were not included in MINS (Devereaux and Szczeklik 2019). Mortality was categorized as cardiovascular or non-cardiovascular mortality. Cardiovascular mortality included death due to MI, heart failure, cardiac arrhythmia, stroke, or vascular causes, and any death without an undisputed non-cardiovascular cause. A total of 125 deaths without apparent non-cardiovascular causes were considered as cardiovascular deaths (Hicks et al. 2015). Medical history was obtained by reviewing the extracted electronic medical records of the preoperative evaluation. The 2014 European Society of Cardiology/Anesthesiology guidelines were used to define high-risk surgeries (Kristensen and Knuuti 2014)^.^

#### Perioperative management and cTn I

Postoperative cTn I follow-up was recommended for moderate- or high-risk surgery or for patients with at least one major cardiovascular risk factor, such as a history of ischemic heart disease; heart failure; stroke, including transient ischemic attack; diabetes mellitus requiring insulin therapy; or chronic kidney disease, based on current guidelines (Kristensen and Knuuti 2014). In patients with minor risk factors, cTn I was measured at the discretion of the attending clinician in older patients or those with recently suspected symptoms of ischemic disease. An automated analyzer (Advia Centaur XP, Siemens Healthcare Diagnostics, Erlangen, Germany) with a highly sensitive immunoassay was used. The lowest limit of detection was 0.006 ng/mL, and 0.04 ng/mL was determined to be the 99th percentile URL by the manufacturer (Mahajan and Jarolim 2011). The same cTn assay and cut-offs were used throughout the study period.

### Statistical analysis

Continuous variables are described as means (standard deviations [SDs]) or medians (interquartile ranges [IQRs]), and categorical variables are expressed as numbers (%). We used parametric or non-parametric tests as appropriate to compare the differences in the baseline characteristics in the preliminary analyses. Outcomes were compared using stratified logistic or Cox regression models. The Fine and Gray model subdistribution hazard model was used to adjust the competing risk. The hazard ratio (HR) or odds ratio (OR) with 95% confidence interval (CI) were reported, and a multivariable analysis for unmatched data was performed using stepwise backward selection with *P* < 0.05 for inclusion of variables and *P* > 0.10 for removal of variables. Kaplan–Meier survival curves were constructed and compared using log-rank tests. Among patients with MINS, men were matched with women using propensity-score-based 1:1 individual caliper matching without case replacement to maximize study power while maintaining a balance in confounding factors between the groups. The following factors contributed to the propensity score: age; diabetes; hypertension; current smoking and/or alcohol consumption; chronic kidney disease; history of ischemic heart disease, heart failure, stroke, arrhythmia, or heart valve disease; active cancer; preoperative care (treatment in the intensive care unit, extracorporeal membranous oxygenation, continuous renal replacement therapy, ventilator support); and intraoperative risk factors (European Society of Cardiology/Anesthesiology surgical high risk, emergency operation, general anesthesia, operation duration, continuous infusion of inotropics, and RBC transfusion) (Table 1). Binary logistic regression was used for propensity score estimation. Variables included as ‘intraoperative risk factors’ and ‘preoperative risk factors’ were selected based on previous research. The caliper width was 0.2 SDs of the logit-transformed propensity score. A balance between the two groups was deemed to be achieved when the absolute standardized mean difference was < 10% and the variance ratio was close to 1.0 for each of the covariates. For sensitivity analyses, we estimated the potential impact of unmeasured confounders. The risks of outcome were compared using Cox or logistic regression models. Regarding sample size, the power of this study was estimated before formal analysis. It was 0.99 in the entire population when the OR of MINS was < 0.8 and 0.99 in the matched population when the HR of 30-day mortality of patients with MINS was < 0.8. All reported *P* values were two-tailed, and statistical significance was set at *P* < 0.05. All statistical analyses were performed using R 3.6.1 (Vienna, Austria; http://www.R-project.org/).

**Table 1:** Baseline characteristics of the entire population

	Entire population			
	Male (*N* = 18,546)	Female (*N* = 14,765)	*P* value	SMD
Age	62.01 (13.4)	62.48 (14.0)	0.002	3.5
Diabetes	10,102 (54.5)	6819 (46.2)	< 0.001	16.6
Hypertension	10,518 (56.7)	8187 (55.4)	0.02	2.5
Current smoking	3015 (16.3)	272 (1.8)	< 0.001	51.9
Current alcohol	5398 (29.1)	1204 (8.2)	< 0.001	55.9
Chronic kidney disease	1195 (6.4)	645 (4.4)	< 0.001	9.2
History of ischemic heart disease	3356 (18.1)	1593 (10.8)	< 0.001	20.9
History of heart failure	407 (2.2)	553 (3.7)	< 0.001	9.1
History of stroke	1365 (7.4)	841 (5.7)	< 0.001	6.7
History of arrhythmia	1382 (7.5)	820 (5.6)	< 0.001	7.7
History of heart valve disease	188 (1.0)	240 (1.6)	< 0.001	5.4
Active cancer	9274 (50.0)	5123 (34.7)	< 0.001	31.4
Preoperative care				
Intensive care unit	768 (4.1)	475 (3.2)	< 0.001	4.9
ECMO	1 (0.0)	0 (0.0)	> 0.99	1
Continuous renal replacement therapy	50 (0.3)	30 (0.2)	0.26	1.4
Ventilator	134 (0.7)	86 (0.6)	0.13	1.7
Operative variables				
ESC/ESA surgical high risk	4737 (25.5)	1978 (13.4)	< 0.001	31
Emergency operation	2668 (14.4)	1963 (13.3)	0.004	3.2
General anesthesia	17,158 (92.5)	11,997 (81.3)	< 0.001	33.8
Operation duration, hours	3.38 (2.27)	2.80 (2.05)	< 0.001	26.8
Continuous infusion of inotropics	5447 (29.4)	4217 (28.6)	0.11	1.8
RBC transfusion	1559 (8.4)	895 (6.1)	< 0.001	9.1

Data are presented as *n* (%) or mean (± standard deviation)

*SMD* standardized mean difference, *ECMO* extracorporeal membranous oxygenation, *RAAS* renin–angiotensin–aldosterone system, *ESC* European society of cardiology, *ESA* European Society of Anaesthesiology, *RBC* red blood cell

## Results

### Incidence of MINS

**Fig. 1:**
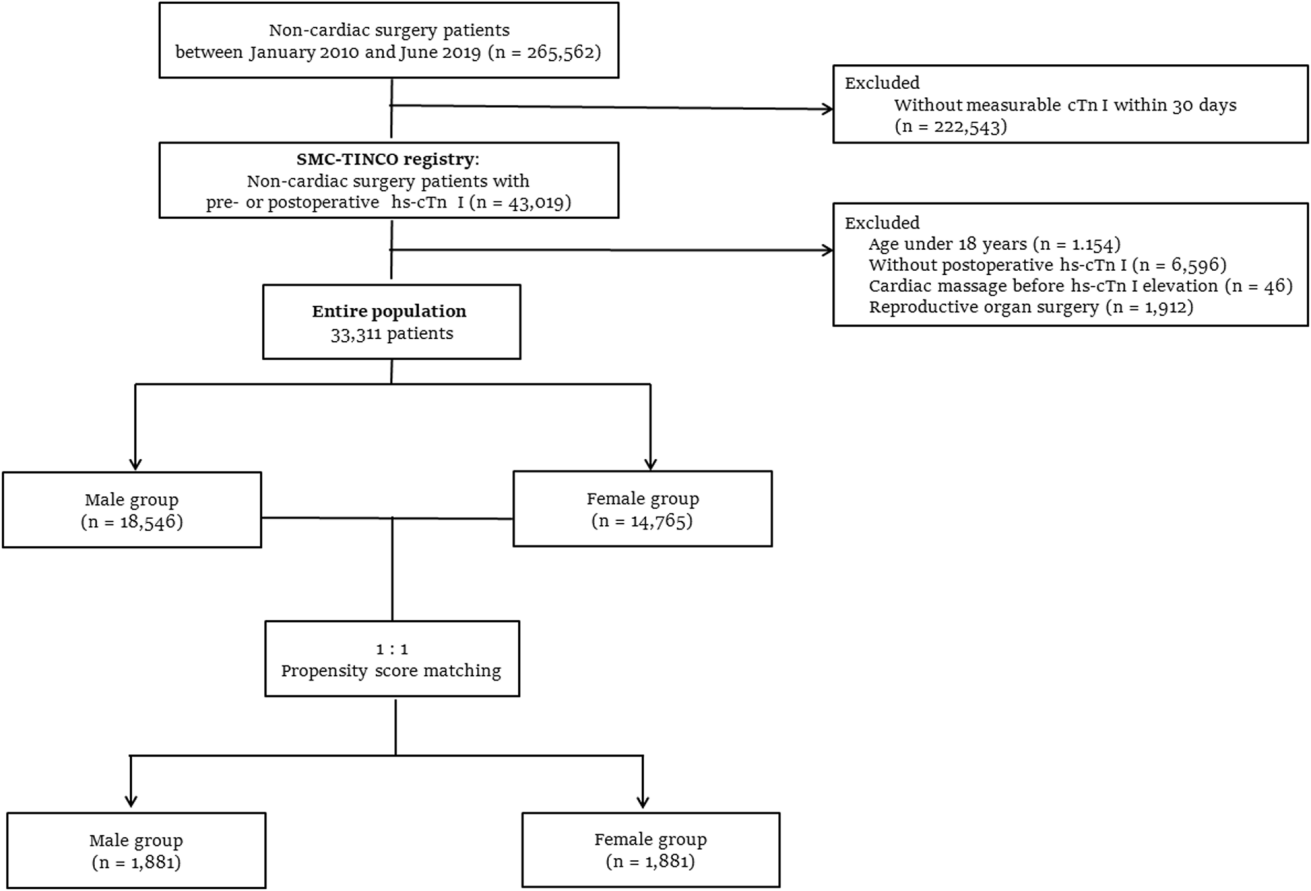
Flow diagram

After excluding 1154 patients under 18 years of age, 6596 patients without postoperative cTn I measurement, 46 patients who received cardiac massage, and 1912 patients who underwent reproductive organ-specific surgery, a total of 33,311 patients remained in this study. The patients were divided into two groups according to sex: 18,546 (55.7%) men and 14,765 (44.3%) women (Fig. 1). Baseline characteristics are summarized in Table 1. Women showed a lower incidence of preoperative comorbidities and intraoperative risk factors. Of the 5594 patients with postoperative cTn elevation, 188 patients had non-ischemic diseases, and the overall incidence of MINS was 16.2% (5406/33,311). There was a significantly lower incidence of MINS among women (17.9% vs. 14.2%; adjusted OR, 0.76; 95% CI, 0.71–0.81; *P* < 0.001). Similarly, 1 year mortality was also lower in women (10.5% vs. 7.0%; HR, 0.66; 95% CI, 0.61–0.71; *P* < 0.001; Table 2). The overall mortality was also lower in women (22.1% vs. 14.0%; HR, 0.63; 95% CI, 0.60–0.67; *P* < 0.001) (Table 2). Women had shorter hospital stays than men (8 days vs. 9 days; *P* < 0.001) (Table 2). The types of surgery are presented in Table S1 in the Electronic Supplementary Material (ESM).

**Table 2:** Clinical outcomes the entire population

	Male (*N* = 18,546)	Female (*N* = 14,765)	Univariate analysis		Multivariate analysis	
			Unadjusted OR/HR (95% CI)	*P* value	Adjusted OR/HR (95% CI)	*P* value
Myocardal injury after non-cardiac surgery^a^	3314 (17.9)	2092 (14.2)	0.76 (0.72–0.81)	< 0.001	0.76 (0.71–0.81)	< 0.001
In-hospital mortality	489 (2.6)	262 (1.8)	0.85 (0.73–0.99)	0.04	0.79 (0.67–0.93)	0.004
30-day mortality	377 (2.0)	227 (1.5)	0.75 (0.64–0.89)	< 0.001	0.65 (0.54–0.77)	< 0.001
Cardiovascular death	86 (0.5)	57 (0.4)	0.83 (0.59–1.16)	0.28	0.83 (0.58–1.20)	0.33
Non-cardiovascular death	291 (1.6)	170 (1.2)	0.73 (0.61–0.88)	0.001	0.60 (0.49–0.74)	< 0.001
1-year mortality	1950 (10.5)	1038 (7.0)	0.66 (0.61–0.71)	< 0.001	0.60 (0.55–0.65)	< 0.001
Cardiovascular death	590 (3.2)	315 (2.1)	0.66 (0.58–0.76)	< 0.001	0.65 (0.56–0.75)	< 0.001
Non-cardiovascular death	1360 (7.3)	723 (4.9)	0.66 (0.60–0.72)	< 0.001	0.58 (0.53–0.64)	< 0.001
Overall mortality	4101 (22.1)	2065 (14.0)	0.62 (0.59–0.65)	< 0.001	0.63 (0.60–0.67)	< 0.001
Cardiovascular death	1745 (9.4)	887 (6.0)	0.62 (0.58–0.68)	< 0.001	0.67 (0.62–0.73)	< 0.001
Non-cardiovascular death	2356 (12.7)	1178 (8.0)	0.62 (0.58–0.66)	< 0.001	0.61 (0.56–0.65)	< 0.001
Hospital stayb	9 (6–15)	8 (6–13)		< 0.001		

Data are presented as *n* (%)

*OR* odds ratio, *HR* hazard ratio, *CI* confidence interval

^a^Presented as OR

^b^Presented as median (IQR)

**Fig. 2:**
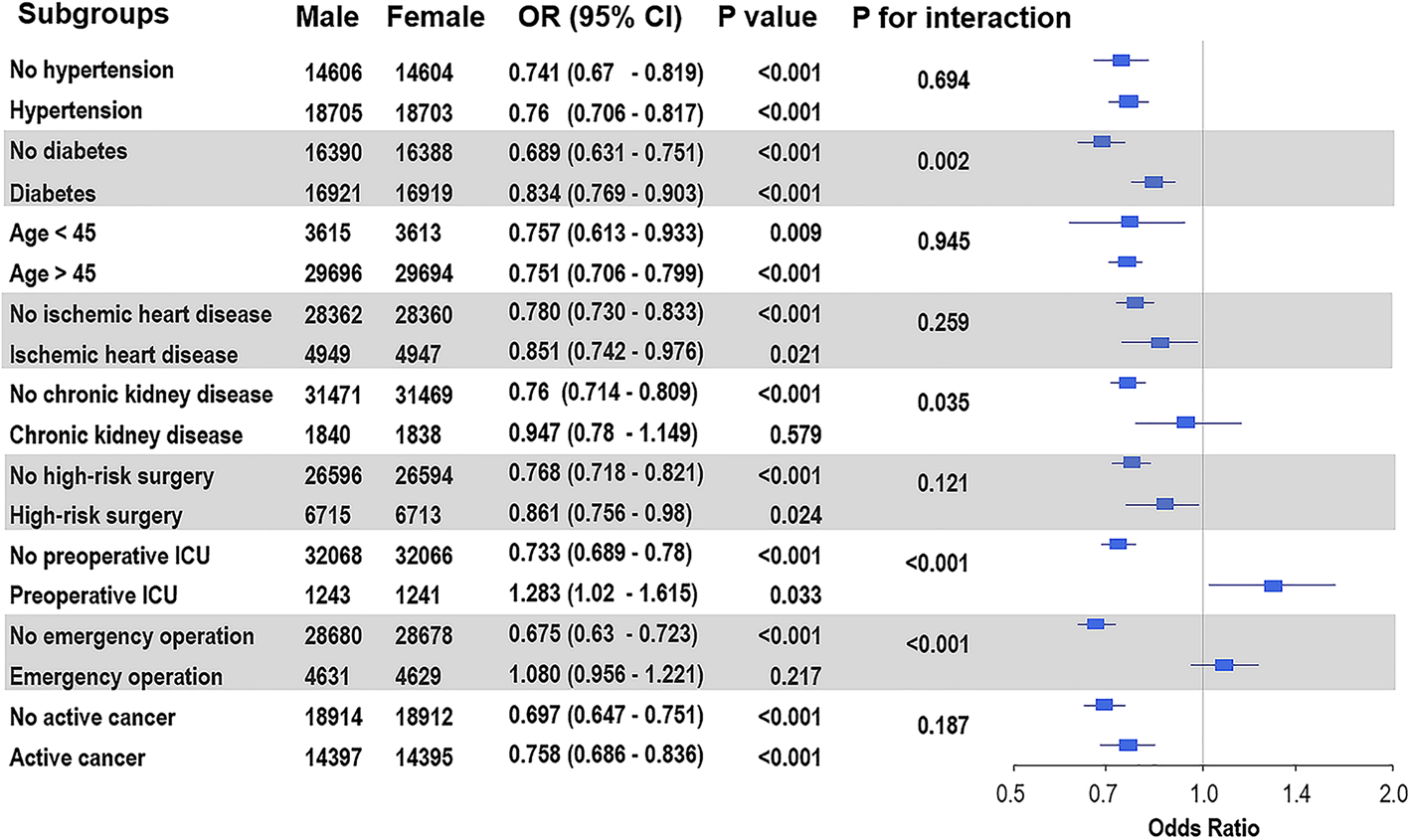
Forest plot for subgroup analysis of the occurrence of myocardial injury after non-cardiac surgery in the entire population

Significant interactions between preoperative intensive care and emergency surgery and MINS were observed (*p* for interaction < 0.001 for preoperative intensive care, < 0.001 for emergency surgery), and the protective effect of the female sex against MINS was limited to patients without preoperative intensive care or those who had undergone emergency surgery (Fig. 2). The significance of the observed association between sex and MINS was also evaluated according to the type of surgery and is presented in Fig. 2. Men showed a significantly higher incidence of MINS in the vascular surgery and urological surgery subgroups, and women showed a higher incidence in the orthopedic surgery, neurosurgery, and non-cardiothoracic surgery subgroups. The sensitivity to the effect of an unmeasured confounder on the observed association was also evaluated assuming that the prevalence of measured confounders was 40%, and the association was significant under all circumstances (Additional file 1: Table S2).

### Mortality after MINS

The total of 5406 adult patients with MINS included 3314 (61.3%) men and 2092 (38.7%) women (Additional file 1: Table S3 in the ESM). In the entire population, the median follow-up duration for 30-day mortality was 30 days (IQR 30–30) in both groups, and all patients without mortality completed 30 days of follow-up. The median duration of overall follow-up was 1.67 years (IQR 0.39–4.03) in men and 1.72 years (IQR 0.44–3.94) in women (*P* = 0.77). The multivariable analysis showed no significant difference in 30-day mortality, but the overall mortality was lower in women (32.7% vs. 26.0%; HR, 0.76; 95% CI, 0.68–0.84; *P* < 0.001) (Table 3).

**Table 3:** Clinical outcomes of the patients with myocardial injury after non-cardiac surgery before and after propensity score matching

Before matching						
Before matching			Univariate analysis		Multivariate analysis	
	Male (*N* = 3314)	Female (*N* = 2092)	Unadjusted /HR (95% CI)	*P* value	Adjusted OR/HR (95% CI)	*P* value
30-day mortality	253 (7.6)	154 (7.4)	0.96 (0.79–1.18)	0.72	0.84 (0.68–1.04)	0.12
Cardiovascular death	59 (1.8)	42 (2.0)	1.13 (0.76–1.68)	0.55	1.05 (0.69–1.61)	0.82
Non-cardiovascular death	194 (5.9)	112 (5.4)	0.91 (0.72–1.15)	0.45	0.79 (0.61–1.01)	0.06
1-year mortality	687 (20.7)	372 (17.8)	0.85 (0.75–0.97)	0.01	0.76 (0.67–0.87)	< 0.001
Cardiovascular death	191 (5.8)	100 (4.8)	0.82 (0.65–1.05)	0.11	0.75 (0.58–0.97)	0.03
Non-cardiovascular death	496 (15.0)	272 (13.0)	0.86 (0.74–0.99)	0.05	0.77 (0.66–0.90)	0.001
Overall mortality	1085 (32.7)	544 (26.0)	0.79 (0.72–0.88)	< 0.001	0.76 (0.68–0.84)	< 0.001
Cardiovascular death	409 (12.3)	196 (9.4)	0.76 (0.64–0.90)	0.002	0.77 (0.64–0.92)	0.004
Non-cardiovascular death	676 (20.4)	348 (16.6)	0.81 (0.71–0.92)	0.002	0.75 (0.65–0.86)	< 0.001
After matching						
	Male (*N* = 1881)	Female (*N* = 1881)	Adjusted HR (95% CI)	*P* value		
30-day mortality	158 (8.4)	130 (6.9)	0.81 (0.64–1.03)	0.09		
Cardiovascular death	36 (1.9)	36 (1.9)	1.0 (0.63–1.58)	0.98		
Non-cardiovascular death	122 (6.5)	94 (5.0)	0.76 (0.58–1.0)	0.05		
1-year mortality	426 (22.6)	319 (17.0)	0.72 (0.63–0.84)	< 0.001		
Cardiovascular death	114 (6.1)	85 (4.5)	0.72 (0.54–0.95)	0.02		
Non-cardiovascular death	312 (16.6)	234 (12.4)	0.72 (0.60–0.85)	0.002		
Overall mortality	621 (33.0)	483 (25.7)	0.75 (0.66–0.84)	< 0.001		
Cardiovascular death	222 (11.8)	178 (9.5)	0.77 (0.63–0.94)	0.01		
Non-cardiovascular death	399 (21.2)	305 (16.2)	0.74 (0.63–0.85)	< 0.001		

Data are presented as *n* (%)

*HR* hazard ratio, *CI* confidence interval

**Fig. 3:**
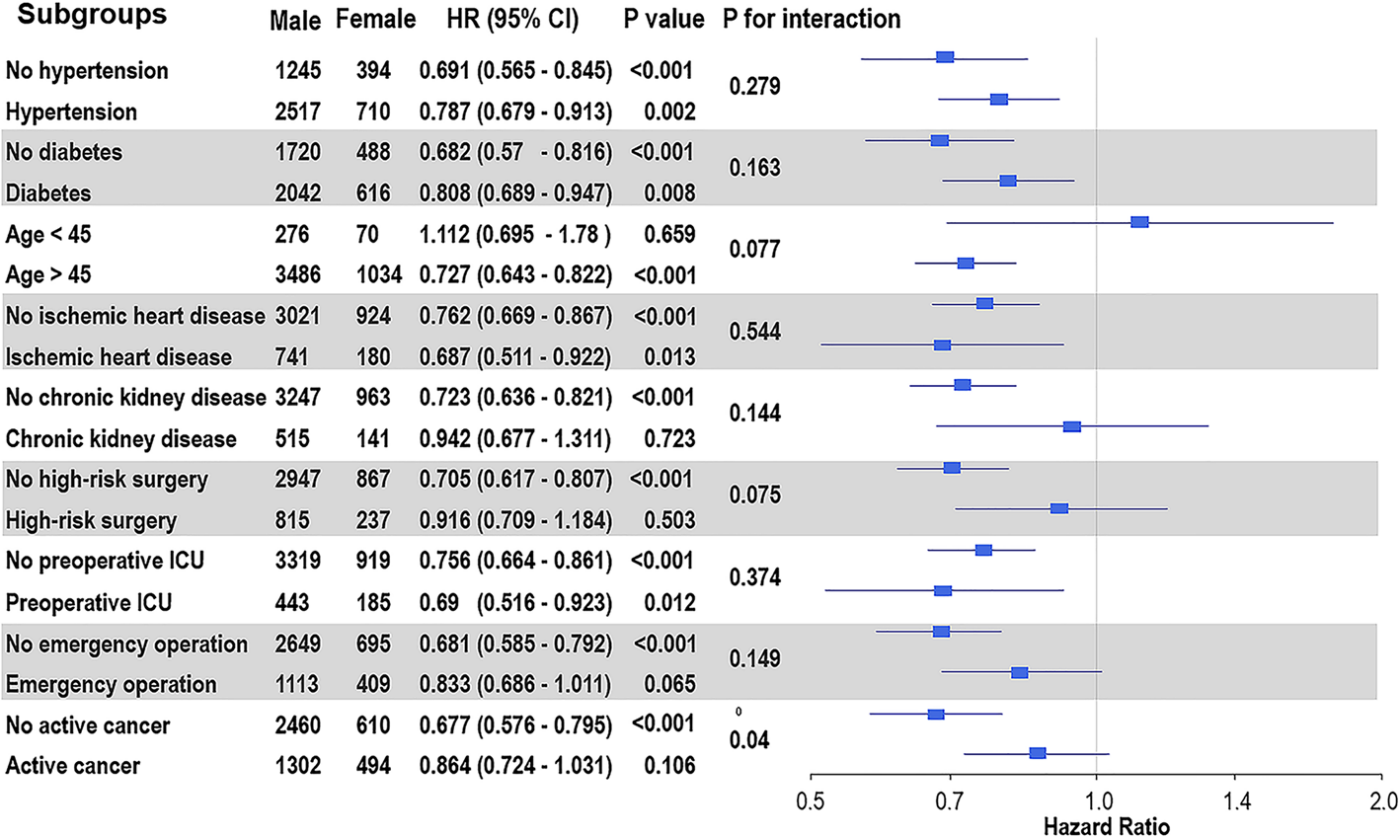
Forest plot for subgroup analysis of survival following myocardial injury after non-cardiac surgery

**Fig. 4:**
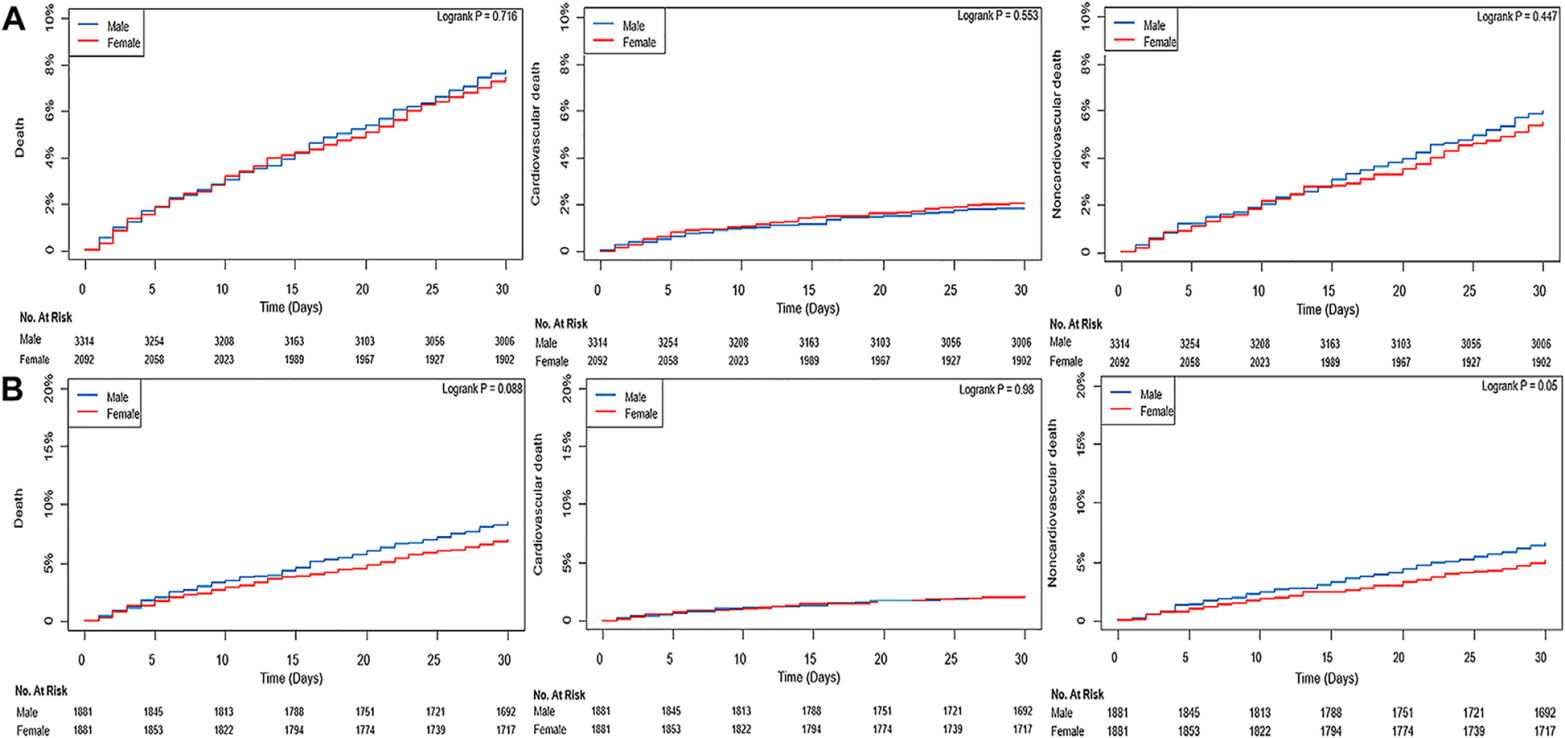
Kaplan–Meier curves for 30-day mortality in the **A** entire population and **B** propensity-score-matched population

After propensity score matching, all covariates were well balanced (Additional file 1: Table S3). The median follow-up duration for 30-day mortality was 30 days (IQR 30–30) in both groups, and the median duration of overall follow-up was 1.50 years (IQR 0.33–3.92) in men and 1.76 years (IQR 0.47–3.96) in women (*P* = 0.02). In the propensity-score-matched population, 30-day mortality was not significantly different; however, during the 1-year follow-up period, women showed a lower risk of mortality irrespective of the cause of death (22.6% vs. 17.0%; HR, 0.72; 95% CI, 0.63–0.84; *P* < 0.001 for all-cause mortality; 6.1% vs. 4.5%; HR, 0.72; 95% CI, 0.54–0.95; *P* = 0.02 for cardiovascular mortality; and 16.6% vs. 12.4%; HR, 0.72; 95% CI, 0.60–0.85; *P* = *0.002* for non-cardiovascular mortality) (Table 3 and Additional file 3: Figure S2). During the overall follow-up period, women also showed a lower risk of mortality irrespective of the cause of death (33.0% vs. 25.7%; HR, 0.75; 95% CI, 0.66–0.84; *P* < 0.001 for all-cause mortality; 11.8% vs. 9.5%; HR, 0.77; 95% CI, 0.63–0.94; *P* = 0.01 for cardiovascular mortality; and 21.2% vs. 16.2%; HR, 0.74; 95% CI, 0.63–0.85; *P* < 0.001 for non-cardiovascular mortality) (Table 3 and Additional file 4: Figure S3). In the subgroup analysis, the observed association between sex and mortality after MINS was not significantly affected by any covariate (Fig. 3). Survival curves for overall and 30-day mortalities are shown in Additional file 4: Figure S3 and Fig. 4, respectively. Cumulative incidence functions and Kaplan–Meier estimates for cardiovascular and non-cardiovascular death are shown in Additional file 5: Figure S4.

## Discussion

This study showed that female sex was independently associated with a lower incidence of MINS and was associated with decreased overall mortality following MINS irrespective of the cause of death. Our findings suggest that sex differences need to be considered in designing future study on and the clinical management of MINS.

A large international study showed increased mortality in patients with MINS (Devereaux et al. 2017). However, unlike postoperative MI, there is ongoing debate on the causal relationship between MINS and mortality and the mechanism of postoperative cTn elevation. A recent study demonstrated that circulating microRNAs associated with cardiac ischemia were universally elevated after surgery, independent of cTn elevation. (May et al. 2020). Therefore, the prognosis of patients with cTn elevation requires a separate investigation of sex differences, as shown in a previous study on ischemic heart disease (Kovacic et al. 2012). Recently, animal and experimental studies have demonstrated better recovery of the heart from reperfusion injury in females (Mendelsohn and Karas 2005; Regitz-Zagrosek and Kararigas 2017). Thus, being female confers survival benefits over being male during the early days of ischemic stress after MI. Moreover, clinical evidence suggests that women with ischemic heart disease have less coronary atherosclerosis and present with better outcomes than men of the same age or with cardiovascular risk factors (Regitz-Zagrosek and Kararigas 2017). In this study, sex differences shown in previous studies were consistently found in patients with MINS who were diagnosed based on cTn elevation. To our knowledge, this is the first report evaluating the clinical impact of sex differences in other cardiovascular diseases, such as the incidence and survival after MINS.

Another notable finding was that the reduced 30-day mortality was mainly driven by non-cardiovascular deaths rather than cardiovascular deaths. Because we used a broad definition for cardiovascular death, the reduction in mortality in this study was related to definite non-cardiovascular causes. This seems to be related to the diverse mechanisms involved in the occurrence of MINS. Previous studies reported that 11–14% cTn elevation after non-cardiac surgery appeared to be combined with non-ischemic causes that should have been excluded for MINS diagnosis (Landesberg and Jaffe 2015; Noordzij et al. 2015). Numerous mechanisms have been proposed for postoperative cTn elevation, and a recent analysis has questioned the diagnosis of cardiac ischemia based on postoperative cTn elevation (May et al. 2020). These results suggest that increased mortality in patients with cTn elevation may be related to a combination of non-ischemic causes rather than myocardial ischemia directly from coronary issues, and the protective effect of the female sex against MINS shown in our study may also be related to a combination of non-ischemic causes. The subgroup analysis, demonstrating that this protective effect vanishes in women with preoperative intensive care or emergency surgery, which may be related to fatal ischemic complications, also supports this explanation.

Because mortality in the entire population was significantly different according to sex, we performed more rigorous adjustments in patients with MINS. After generating the matched population using propensity score, mortalities during the first year and the overall follow-up periods were consistently lower in women. Owing to the complex etiologies of MINS, a full explanation of these results remains challenging; however, a clue can be inferred from a previous autopsy study showing that fatal cases of perioperative MI are specifically related to coronary plaque rupture rather than oxygen demand/supply mismatch (Cohen and Aretz 1999). Moreover, there is a difference in the type of ischemic heart disease that each sex is prone to develop. While men most frequently suffer from occlusive coronary artery disease, non-obstructive coronary artery disease or microvascular dysfunction are more frequently seen in women (Regitz-Zagrosek and Kararigas 2017). Therefore, the higher incidence of occlusive coronary artery disease in men could have resulted in significantly different mortality rates.

## Limitations

The present study has several limitations. First, our data were obtained from a Korean population who underwent surgeries at a single center. Our data may not be generalizable to populations in other countries. Additionally, the median follow-up duration was relatively short; therefore, our results might be different for longer periods. For outcomes, we only compared MINS, mortality, and length of hospital stay, and the incidence of other morbidities may show different results. Second, although rigorous analysis to adjust for confounders was conducted, we could only correct for known or measured confounders. A bias due to unmeasured variables can exist. In addition, patients undergoing sex-specific procedures were not included in the analysis, and our results might differ according to the type of surgery. Third, previous studies demonstrated a trend for higher values of URL in men, leading to a recommendation for sex-dependent URL of the cTn assay in the future (Gore et al. 2014; Thygesen et al. 2018). However, the authors of the present study set universal URLs for both sexes. The clinical efficacy of applying different cut-off values according to sex in the diagnosis of MINS needs to be evaluated in future studies. Lastly, despite an institutional protocol, cTn I measurements were not routinely performed. Cardiac troponin I was measured in patients with a certain cardiovascular risk; consequently, there was a possibility of selection bias. Selection bias is the most significant barrier, and the success of the study mainly depends on how efficiently the heterogeneity between groups is controlled. Group homogeneity significantly improved in the course of matching. Large multicenter studies in various countries are needed to generalize our findings. Despite these limitations, this is the first study to show an association between sex and mortality in patients with MINS.

## Conclusion

Women showed a lower incidence of MINS than men, and among patients with MINS, women showed a favorable survival rate after adjustment. Continuous efforts are warranted to improve the sex discrepancies related to MINS.

## Supplementary Information

**Additional file 1: Table S1.** Types of surgery. **Table S2.** Sensitivity analysis of the effect of an unmeasured confounder on odds ratio of female for myocardial injury after noncardiac surgery. **Table S3.** Baseline characteristics of the patients with myocardial injury after noncardiac surgery.

**Additional file 2: Figure S1.** Forest plot for subgroup analysis (type of surgery) of the occurrence of myocardial injury after non-cardiac surgery in the entire population.

**Additional file 3: Figure S2.** Kaplan-Meier curves for 1 year mortality in the (A) entire population and (B) propensity-score-matched population.

**Additional file 4: Figure S3.** Kaplan-Meier curves for overall mortality in the (A) entire population and (B) propensity-score-matched population.

**Additional file 5: Figure S4.** Cumulative incidence functions and Kaplan–Meier estimates for cardiovascular and non-cardiovascular deaths.

## Data Availability

The datasets used and/or analyzed during the current study are available from the corresponding author upon reasonable request.

## Abbreviations

CKD: Chronic kidney disease
MI: Myocardial infarction
MINS: Myocardial injury after non-cardiac surgery
cTn: Cardiac troponin
HR: Hazard ratio
CI: Confidence interval
IQR: Interquartile range
