# Supplementary material for: Sex differences in myocardial injury after non-cardiac surgery and postoperative mortality
Source: Perioper Med (Lond). 2023 Mar 16;12:7. doi: 10.1186/s13741-023-00294-3 (PMC10018929; doi:10.1186/s13741-023-00294-3)
Supplement: Supplementary file 1 — Additional file 1: Table S1. Types of surgery. Table S2. Sensitivity analysis of the effect of an unmeasured confounder on odds ratio of female for myocardial injury after noncardiac surgery. Table S3. Baseline characteristics of the patients with myocardial injury after noncardiac surgery. [file 13741_2023_294_MOESM1_ESM.docx]

**eTable 1** Types of surgery

|  | Male  (*N* = 18546) | Female  (*N* = 14765) |
| --- | --- | --- |
| Vascular | 1672 (9.0) | 432 (2.9) |
| Orthopediatric | 1373 (7.4) | 3477 (23.5) |
| Neuro | 3469 (18.7) | 4469 (30.3) |
| Breast or Endo | 237 (1.3) | 327 (2.2) |
| Plastic or Otolaryngeal or Eye | 755 (4.1) | 549 (3.7) |
| Transplantation | 773 (4.2) | 358 (2.4) |
| Urology | 921 (5.0) | 464 (3.1) |
| Gastrointestinal | 6928 (37.4) | 3888 (26.3) |
| Noncardiac thoracic | 2351 (12.7) | 763 (5.2) |
| Others | 67 (0.4) | 38 (0.3) |

**eTable 2** Sensitivity analysis of the effect of an unmeasured confounder on odds ratio of female for myocardial injury after noncardiac surgery

|  |  | OR*_ZY_*_\|_*_X_* | | | | | |
| --- | --- | --- | --- | --- | --- | --- | --- |
|  |  | 1.5 | 2 | 2.5 | 3 | 3.5 | 4 |
| OR_zx_ | 0.3 | 0.84 (0.78-0.90) | 0.92 (0.86-0.98) | 0.99 (0.92-1.06) | 1.05 (0.98-1.12) | 1.09 (1.02-1.17) | 1.14 (1.02-1.17) |
|  | 0.4 | 0.82 (0.77-0.88) | 0.88 (0.82-0.94) | 0.92 (0.86-0.99) | 0.97 (0.90-1.04) | 1.00 (0.93-1.07) | 1.03 (0.96-1.11) |
|  | 0.5 | 0.81 (0.76-0.87) | 0.85 (0.79-0.91) | 0.88 (0.83-0.95) | 0.91 (0.85-0.97) | 0.93 (0.87-1.00) | 0.95 (0.89-1.02) |
|  | 0.6 | 0.80 (0.75-0.85) | 0.83 (0.77-0.88) | 0.85 (0.79-0.91) | 0.87 (0.81-0.93) | 0.88 (0.82-0.94) | 0.89 (0.83-0.95) |
|  | 0.7 | 0.79 (0.74-0.84) | 0.81 (0.75-0.86) | 0.82 (0.77-0.88) | 0.84 (0.78-0.89) | 0.83 (0.78-0.98) | 0.84 (0.79-0.90) |

Prevalence of unmeasured confounder = 40%.

Numbers represent HRs (including 95% CIs).

*OR* odds ratio, *X* dichotomous exposure measure, *y* dichotomous outcome measure, *z* potential dichotomous confounder

OR_ZX_ indicates the association (OR) between the unmeasured confounder and female.

OR_ZY|X_ indicates the association (OR) between the unmeasured confounder and myocardial injury after noncardiac surgery.

**eTable 3** Baseline characteristics of the patients with myocardial injury after noncardiac surgery

|  | **Entire population** | | | | **Propensity score matched population** | | | |
| --- | --- | --- | --- | --- | --- | --- | --- | --- |
|  | **Male**  **(*N* = 3314)** | **Female**  **(*N* = 2092)** | ***p* value** | **SMD**  **(%)** | **Male**  **(*N* = 1881)** | **Female**  **(*N* = 1881)** | ***p* value** | **SMD**  **(%)** |
| Age | 65.16 (13.1) | 66.50 (14.3) | <0.001 | 9.8 | 65.82 (13.51) | 66.45 (14.26) | 0.17 | 4.5 |
| Diabetes | 1888 (57.0) | 1099 (52.5) | 0.002 | 8.9 | 1033 (54.9) | 1009 (53.6) | 0.45 | 2.6 |
| Hypertension | 2229 (67.3) | 1399 (66.9) | 0.79 | 0.8 | 1237 (65.8) | 1280 (68.0) | 0.15 | 4.9 |
| Current smoking | 454 (13.7) | 49 (2.3) | <0.001 | 42.8 | 51 (2.7) | 49 (2.6) | 0.92 | 0.7 |
| Current alcohol | 680 (20.5) | 114 (5.4) | <0.001 | 46.0 | 130 (6.9) | 114 (6.1) | 0.32 | 3.5 |
| Chronic kidney disease | 497 (15.0) | 260 (12.4) | 0.01 | 7.5 | 262 (13.9) | 253 (13.5) | 0.7 | 1.4 |
| History of ischemic heart disease | 898 (27.1) | 379 (18.1) | <0.001 | 21.6 | 368 (19.6) | 373 (19.8) | 0.87 | 0.7 |
| History of heart failure | 108 (3.3) | 92 (4.4) | 0.04 | 5.9 | 74 (3.9) | 88 (4.7) | 0.3 | 3.7 |
| History of stroke | 318 (9.6) | 196 (9.4) | 0.82 | 0.8 | 185 (9.8) | 175 (9.3) | 0.62 | 1.8 |
| History of arrhythmia | 332 (10.0) | 224 (10.7) | 0.44 | 2.3 | 199 (10.6) | 207 (11.0) | 0.71 | 1.4 |
| History of heart valve disease | 46 (1.4) | 62 (3.0) | <0.001 | 10.8 | 35 (1.9) | 53 (2.8) | 0.07 | 6.3 |
| Active cancer | 1441 (43.5) | 636 (30.4) | <0.001 | 27.4 | 670 (35.6) | 632 (33.6) | 0.21 | 4.2 |
| Preoperative care |  |  |  |  |  |  |  |  |
| Intensive care unit | 353 (10.7) | 250 (12.0) | 0.15 | 4.1 | 219 (11.6) | 224 (11.9) | 0.84 | 0.8 |
| ECMO | 1 (0.0) | 0 (0) | >0.99 | 2.5 | 0 | 0 | >0.99 | <0.1 |
| Continuous renal replacement therapy | 38 (1.1) | 24 (1.1) | >0.99 | <0.1 | 21 (1.1) | 22 (1.2) | >0.99 | 0.5 |
| Ventilator | 71 (2.1) | 63 (3.0) | 0.06 | 5.5 | 46 (2.4) | 56 (3.0) | 0.37 | 3.3 |
| Operative variables |  |  |  |  |  |  |  |  |
| ESC/ESA surgical high risk | 1040 (31.4) | 385 (18.4) | <0.001 | 30.4 | 432 (23.0) | 383 (20.4) | 0.06 | 6.3 |
| Emergency operation | 857 (25.9) | 667 (31.9) | <0.001 | 13.3 | 534 (28.4) | 579 (30.8) | 0.12 | 5.2 |
| General anesthesia | 2978 (89.9) | 1694 (81.0) | <0.001 | 25.4 | 1619 (86.1) | 1560 (82.9) | 0.01 | 8.7 |
| Operation duration, hours | 3.73 (2.77) | 3.14 (2.71) | <0.001 | 21.3 | 3.46 (2.76) | 3.29 (2.78) | 0.06 | 6.1 |
| Continuous infusion of inotropics | 1483 (44.7) | 780 (37.3) | <0.001 | 15.2 | 766 (40.7) | 731 (38.9) | 0.26 | 3.8 |
| RBC transfusion | 532 (16.1) | 282 (13.5) | 0.01 | 7.3 | 309 (16.4) | 272 (14.5) | 0.1 | 5.4 |

Data are presented as *n* (%) or mean (±standard deviation).

*SMD* standardized mean difference, *ECMO* extracorporeal membranous oxygenation, *RAAS* renin-angiotensin-aldosterone system, *ESC* European society of cardiology, *ESA* European Society of Anaesthesiology, *RBC* red blood cell
