# Supplementary figures and images for: Sex differences in myocardial injury after non-cardiac surgery and postoperative mortality
Source: Perioper Med (Lond). 2023 Mar 16;12:7. doi: 10.1186/s13741-023-00294-3 (PMC10018929; doi:10.1186/s13741-023-00294-3)

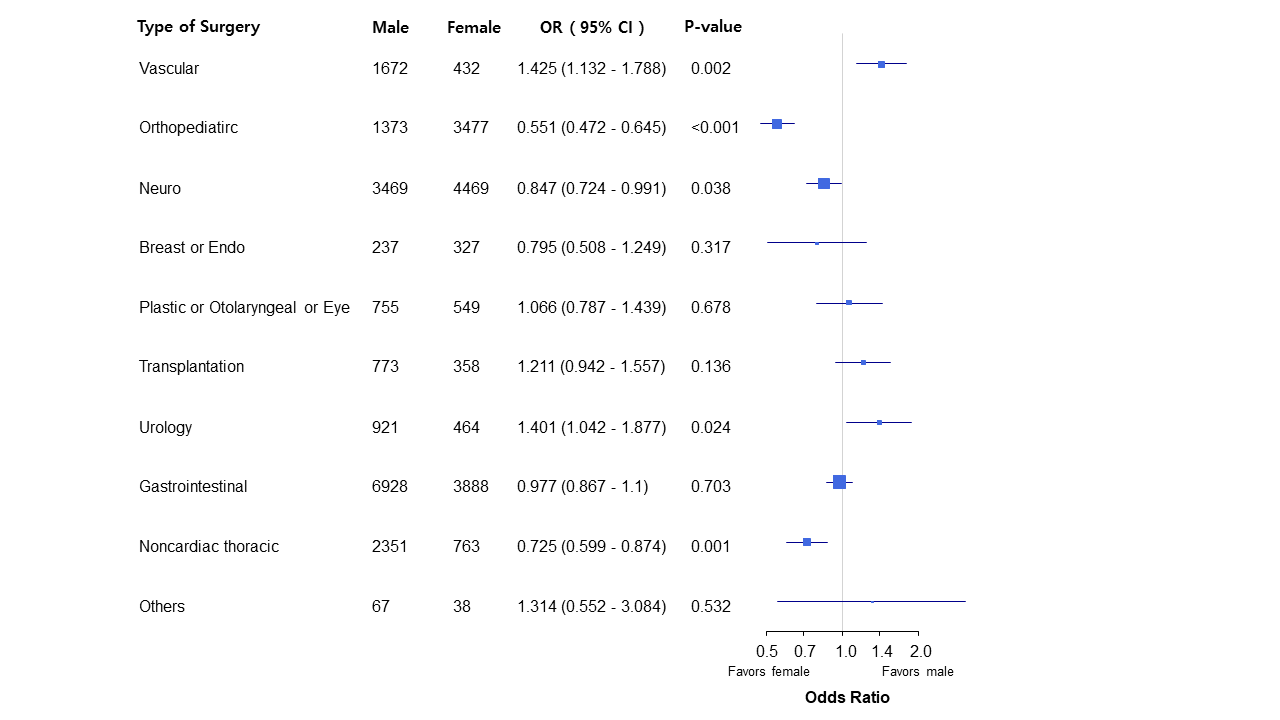

Supplement: Supplementary file 2 — Additional file 2: Figure S1. Forest plot for subgroup analysis (type of surgery) of the occurrence of myocardial injury after non-cardiac surgery in the entire population. [file 13741_2023_294_MOESM2_ESM.tif]

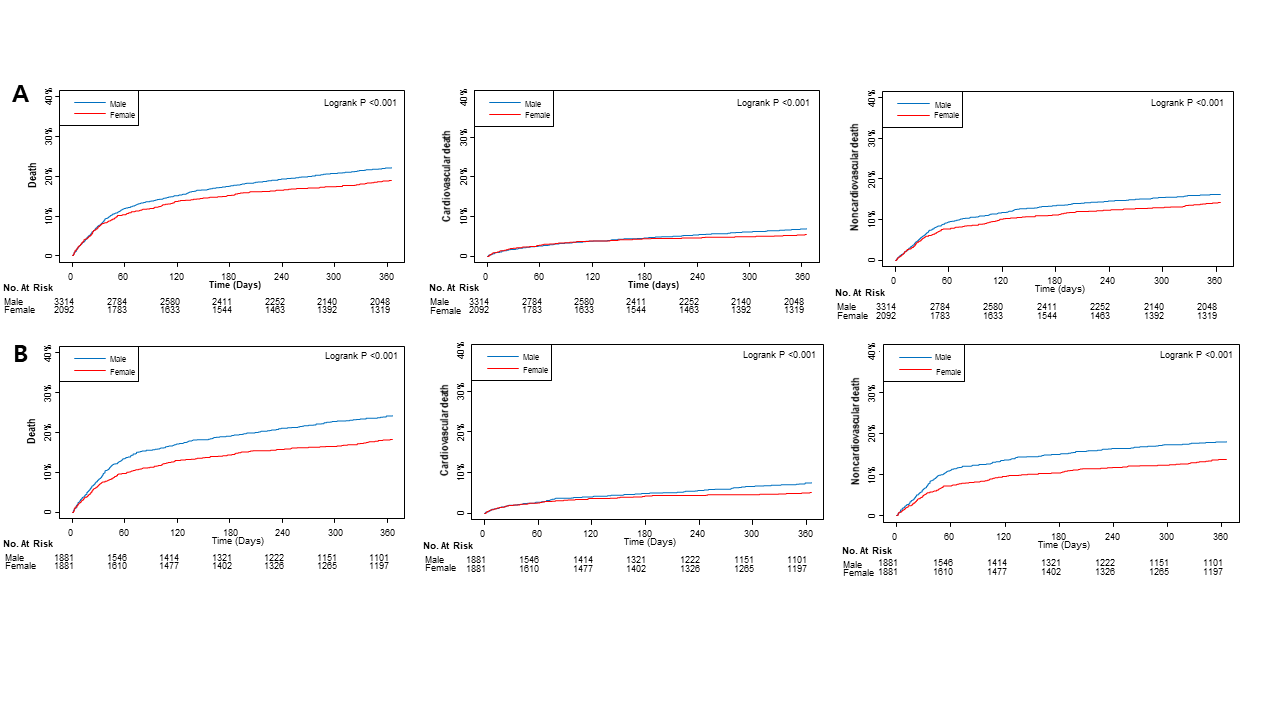

Supplement: Supplementary file 3 — Additional file 3: Figure S2. Kaplan-Meier curves for 1 year mortality in the (A) entire population and (B) propensity-score-matched population. [file 13741_2023_294_MOESM3_ESM.tif]

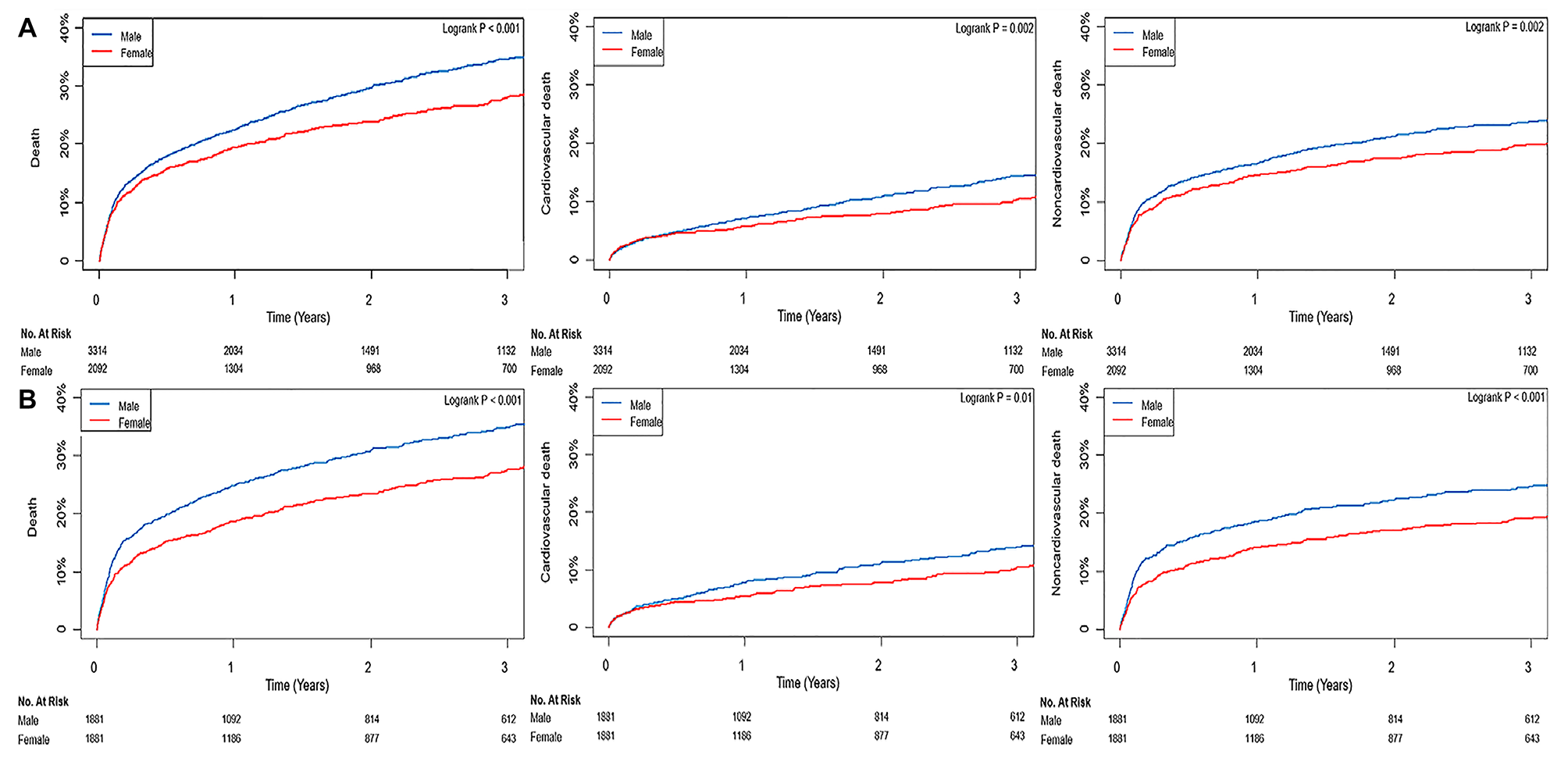

Supplement: Supplementary file 4 — Additional file 4: Figure S3. Kaplan-Meier curves for overall mortality in the (A) entire population and (B) propensity-score-matched population. [file 13741_2023_294_MOESM4_ESM.tif]

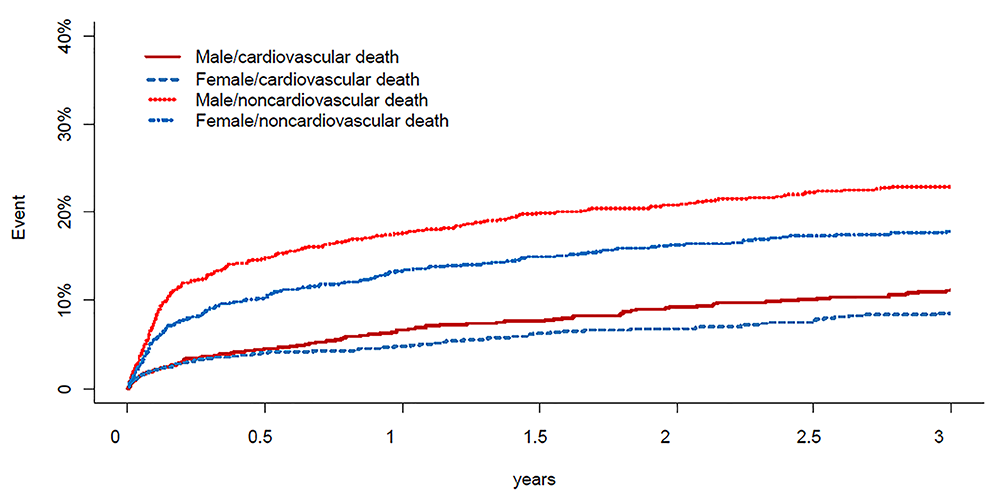

Supplement: Supplementary file 5 — Additional file 5: Figure S4. Cumulative incidence functions and Kaplan–Meier estimates for cardiovascular and non-cardiovascular deaths. [file 13741_2023_294_MOESM5_ESM.tif]
